# Measuring ENAP interventions for small and/or sick newborns in routine health information systems: indicators and considerations from a WHO expert consultation

**DOI:** 10.7189/jogh.15.04134

**Published:** 2025-05-09

**Authors:** WHO expert consultative group on small and/or sick newborn indicators for Every Newborn Action Plan, Samira Aboubaker, Jalemba Aluvaala, Shabina Ariff, Atnafu G Asfaw, KC Ashish, Adejumoke I Ayede, Rajiv Bahl, Anne-Marie Bergh, Helen Brotherton, Nathalie Charpak, Harish Chellani, Msandeni Chiume, Louise Tina Day, Ayesha De Costa, Joseph De Graft-Johnson, Socorro De Leon-Mendoza, Angela Dramowski, Karen Edmond, Shams El Arifeen, Hege Ersdal, Abiy Estifanos, Ute Feucht, Shivaprasad Goudar, Tanya Guenther, Gagan Gupta, Tedbabe Degefie Hailegebriel, Neena Khadka, Rajesh Khanna, Tina Lavin, Joy E Lawn, Marzia Lazzerini, Ornella Lincetto, Carolyn MacLennan, Sarmila Mazumder, Elizabeth McClure, Melissa M Medvedev, Elizabeth M Molyneux, Allisyn C Moran, Susan Niermeyer, Yasir B Nisar, Humphreys Nsona, Shamim A Qazi, Zahida Qureshi, Ahmed E Rahman, Siddharth Ramji, Suman PN Rao, Jennifer Requejo, Harriet Ruysen, Jeeva Sankar, Yirdachew Semu, Sharad Sharma, He Tang, Xu Tao, Gregory C Valentine, Lara ME Vaz, Steve Wall, Robinson D Wammanda, Abadi L Welderufael, Priscilla Wobil, Teshome Woldehanna, Khalid Yunis

## Abstract

**Background:**

Current trends indicate 63 low- and middle-income countries (LMICs) are not on track to achieve the 2030 Sustainable Development Goal 3.2 target of a neonatal mortality rate ≤12 per 1000 live births. The Every Newborn Action Plan (ENAP) prioritised four life-saving interventions for small and/or sick newborns (SSN) in health facilities: neonatal resuscitation, kangaroo mother care, antibiotic treatment of possible serious bacterial infections, and antenatal corticosteroids for women at risk of preterm birth at <34 weeks of gestation. Limited indicator reporting on the use of these interventions in routine health information systems (RHIS) is a barrier to scaling up SSN care.

**Methods:**

The World Health Organization (WHO) led a multi-step process to agree coverage indicators for the four SSN interventions, which included a rapid review of existing research and programme reports; expert consultation to review available evidence, deliberate and propose coverage indicators, assess feasibility in RHIS, and identify research gaps.

**Results:**

Expert working groups discussed and recommended definitions for each of the four coverage indicators. After considering feasibility and challenges, potential sources of data for each indicator were appraised. Data for these indicators is not always routinely collected in registers, requiring information from clinical case records, which can be challenging in resource-constrained health systems. The proposed indicators were also assessed against established indicator assessment criteria. The need for testing the indicators was emphasised and other research gaps were also identified.

**Conclusions:**

Reporting and monitoring the life-saving SSN interventions in routine health information systems (RHIS) is crucial for improving newborn care in LMICs. Urgent consideration must be given to how this data can be collected from health facilities and subsequently reported in RHIS. Improved RHIS measures for these interventions will enable programme managers and policy makers to scale up their use, accelerating reductions in preventable neonatal morbidity and mortality.

Globally, an estimated 2.3 million children die in the newborn period (first 28 days of life) each year, with the majority of deaths (80%) occurring in South Asia and sub-Saharan Africa [1]. Current trends indicate that at least 50 low- and middle-income countries (LMICs) are not on track to achieve the Sustainable Development Goal (SDG) 3.2 target of a neonatal mortality rate ≤12 per 1000 live births by 2030 [2].

Two decades ago, there was a focus on scaling up basic preventative and promotive newborn care and services (hygiene, breastfeeding) at community and first-level facilities [3,4]. This was followed by a scale-up of essential newborn care (immediate care at birth and resuscitation, thermal care, initiation of breastfeeding, prevention of infection, and recognition of danger signs). A recent publication by the World Health Organization (WHO) and the United Nations Children’s Fund (UNICEF) suggests that providing special care for small and/ or sick newborns in district-level hospitals represents the next frontier for accelerating the rate of reduction in neonatal mortality [5].

The global Every Newborn Action Plan (ENAP) launched in June 2014 provides a road map of strategic actions for ending preventable newborn mortality and stillbirth, and was endorsed by the World Health Assembly that year [6]. A WHO technical consultation on newborn health indicators was held in 2014 to measure progress under ENAP towards goals in neonatal mortality reduction for the ENAP metrics [7]. The group, after a detailed process, prioritised 10 core indicators (**Table 1**) to measure the impact and coverage of interventions for all mothers and newborns, and the implementation of interventions for newborns at risk. This last group comprises four indicators to measure four life-saving interventions for small and/or sick newborns (SSN), *i.e.* newborns who are born too early, too small, or who become sick in the first few weeks of life. These include neonatal resuscitation, kangaroo mother care (KMC), antibiotic treatment of possible serious bacterial infections (PSBI), and antenatal corticosteroids (ACS) for women with a high likelihood of preterm birth between 24 weeks to 34 weeks gestation [7,8]. Strong evidence (including randomised trials) for the effect of these interventions has been generated, and all four interventions are recommended in WHO guidelines [9–11]. However, clear definitions for these indicators were not provided in the 2015 consultation. Since then, several studies have assessed the reporting and performance of coverage indicators for SSN care in LMICs [12–15] in routine health information systems (RHIS) using different indicator definitions. There is no global consensus, however, on the most feasible indicator definitions for measuring these four SSN interventions with a view to reporting these in RHIS in LMICs.

**Table 1:** Every Newborn Action Plan ten core indicators [8]

Status		Core ENAP Indicators
Definitions clear, but quantity and consistency of data lacking	Impact	1. Maternal mortality ratio
		2. Stillbirth rate
		3. Neonatal mortality rate
Contact points definitions clear, but data on content of care are lacking	Coverage: care for all mothers and newborns	4. Skilled attendant at birth
		5. Early postnatal care for mothers and babies
		6. Essential newborn care
Gaps in definitions, requiring validation and feasibility testing for HMIS use	Coverage: care for newborns at risk or with complications (specific treatment interventions)	7. Antenatal corticosteroid use
		8. Neonatal resuscitation
		9. Kangaroo mother care
		10. Treatment of serious neonatal infections

ENAP – Every Newborn Action Plan, HMIS – health management information system

Given the critical role of SSN care in reducing neonatal mortality, the WHO and UNICEF recommend tracking the coverage of these interventions in RHIS [16,17]. Routine data use in high-mortality settings has been shown to improve coverage and quality of care [18,19].

In 2022, the joint Every Newborn Action Plan – Ending Preventable Maternal Mortality (ENAP-EMPP) Measurement Improvement Roadmap (2023–25) was developed to strengthen measurement linked to ENAP-EPMM targets and priorities [20]. The WHO secretariat, guided by a Technical Steering Group, initiated a multi-step process, including convening an expert consultative group to review the definitions of the four SSN coverage indicators, assess measurement feasibility in RHIS, and identify research gaps.

Here we aim to describe this multi-step process, present the indicators for SSN suggested by the expert groups, and discuss important considerations related to each indicator, as deliberated by the expert groups that resulted in the proposed indicator.

## METHODS

We undertook a multi-step process to review and identify SSN indicators to inform measurement in RHIS. Step one involved a rapid review of peer-reviewed research and programme reports related to indicators for the four SSN interventions, published between the launch of the 2015 ENAP Measurement Improvement Roadmap [21] and September 2021. Specifically, we identified studies and programme reports that had developed and reported on coverage or other indicators related to SSN (such as duration, service readiness) and organised their details in a matrix according to each SSN intervention (**Online Supplementary Document**). The matrix included information on the indicator study design, setting, and metadata related to the proposed indicator.

Step two involved a WHO-convened multi-stakeholder consultation to review the available evidence found in the rapid review, deliberate and propose feasible SSN indicators for potential use in RHIS, and identify research gaps to improve data availability and quality. Between July 2021 and July 2022, the WHO coordinated a series of consultative meetings with over 80 global experts who were divided into four subgroups, one for each of the four indicators. Experts from diverse backgrounds, including from high neonatal mortality settings, as well as various disciplines (such as obstetrics, midwifery, neonatal medicine and measurement) and institutions (such as ministries of health, academia, professional bodies, multilateral, and non-governmental organisations), were invited to participate.

In August 2021, a preliminary joint meeting was held to discuss the background, objectives, and tasks of the four subgroups. Subsequently, each subgroup convened separately for a series of consultations over the year. The subgroups primarily assessed the following criteria: existing indicator metadata, including the numerator (*e.g.* number receiving the intervention) and the denominator (*e.g.* the total population in need); potential alternatives (particularly for denominators); and the feasibility of measurement within existing RHIS in LMICs.

The third step involved obtaining consensus on the proposed indicators. Each subgroup scored the proposed indicators against established ‘good indicator’ criteria [22] and reached a consensus through open dialogue on a proposed coverage indicator. Additionally, each subgroup suggested areas for further research to improve the measurement of the respective SSN indicator. These suggestions stemmed from points of debate within the group and identified areas in the literature requiring further clarification.

In the final consensus step, the WHO convened a joint review meeting involving the four subgroups and representatives from the Mother and Newborn Information for Tracking Outcomes and Results (MoNITOR), a WHO technical advisory group, to provide additional advice and comment. Subgroup reports were shared at the meeting for feedback, following which the WHO secretariat and the Technical Steering Group agreed upon the final indicator metadata for potential RHIS measurement.

## RESULTS

The proposed SSN intervention indicators and potential sources of data are presented in **Table 2**. Below we outline the main considerations from the subgroups for the recommended indicators.

**Table 2:** Proposed SSN Indicators, numerators and denominators and potential data sources

		Numerator		Denominator	
**Neonatal resuscitation**	**Definition**	Number of newborns receiving positive pressure ventilation at birth.		Total number of newborns not breathing well at birth.*	
	**Source**	Individual patient case records	Labour and delivery register	Individual patient case records	Labour and delivery register
**KMC**	**Definition**	Number of admitted newborns with a birthweight <2500 g who were initiated on KMC (placed in the kangaroo position) anywhere in the facility.†		Number of admitted newborns with a birthweight <2500 g anywhere in the facility.	
	**Source**	Individual patient case records	Neonatal or KMC register	Individual patient case records	Neonatal or KMC register
**Antibiotic treatment for neonatal infection**	**Definition**	Number of neonates identified as cases of possible serious bacterial infection (critical illness or clinically severe infection) in outpatient settings or clinically suspected sepsis in inpatient settings, who received at least two days of appropriate injectable antibiotics.‡		Number of neonates (0–28 days) identified as cases of possible serious bacterial infection (critical illness or clinically severe infection) in outpatient settings or clinically suspected sepsis in inpatient settings.	
	**Source**	Individual patient case records	Neonatal register	Individual patient case records	Neonatal register
**ACS use**	**Definition**	Number of women who delivered between 24 and 34 weeks of gestational age who received at least one dose of ACS§		Number of women who delivered between 24 and 34 weeks of gestational age	
	**Source**	Individual patient case records	Labour and delivery or special register	Individual patient case records	Labour and delivery or special register

ACS – antenatal corticosteroids, KMC – kangaroo mother care

*Excluding macerated stillbirths.

†Initiated on KMC means placed in the ‘kangaroo position’, defined as baby placed in an upright position, in direct skin-to-skin contact on the mother's chest, secured in place using a wrap and/or binder.

‡PSBI means possible serious bacterial infection, *i.e.* one or more of the following clinical signs: severe chest indrawing; high body temperature (38°C or above); fast breathing (60 breaths per minute or more) in infants <7 days; not feeding well; low body temperature (less than 35.5°C); movement only when stimulated) or critical illness (one or more of the following clinical signs: convulsions; not able to feed at all; no movement on stimulation).

§Gestational age estimate using best obstetric estimate, but not post-natal gestational age assessment.

### Neonatal resuscitation – indicator and measurement considerations

Through the rapid review, we identified 13 studies that reported indicators for resuscitation at birth, mostly from sub-Saharan Africa and Southeast Asia (**Online Supplementary Document**). We found no studies that evaluated resuscitation indicators using the criteria for assessing a ‘good indicator’. Various definitions of resuscitation were also used, including receipt of bag and mask ventilation [23–26], drying and stimulation [24], head positioning [24,26], and suctioning [25,27].

Resuscitation is recommended for all babies who do not breathe after birth and includes multiple actions as part of a clinical algorithm [28–30]. The subgroup acknowledged that while the ‘bag and mask’ is commonly used for ventilation during resuscitation, there are several other devices for positive-pressure ventilation (PPV) such as ‘T-pieces’. Therefore, they proposed a broader numerator covering all forms of PPV.

The subgroup recommended ‘total number of infants not breathing well’ as the denominator to reflect the population at risk who may require ventilation. This denominator aligns with the sequence of immediate care after birth in the WHO essential newborn care (ENC) algorithms [29], where infants who are not breathing well are given PPV. The subgroup also recognised there are many definitions of ‘not breathing well’, and for this indicator, they defined it in line with the WHO ENC algorithm: infants breathing shallowly, gasping, or not breathing at all. They emphasised that the denominator should exclude macerated stillbirths, meaning that resuscitation efforts should not be made for stillborn infants who show obvious signs of maceration.

### Kangaroo mother care – indicator and measurement considerations

Various studies and reports assessing KMC coverage indicators used different definitions for denominators. Five studies reported the proportion of low birthweight newborns or all newborns initiated on KMC in the facility [23,31–35]. The proportion of babies who received KMC who were discharged alive or discharged after meeting adequate weight criteria were also reported in three studies [31,33,36], although these indicators reflect KMC outcomes rather than KMC coverage. Other studies reported on the proportions of newborns referred out of facility for KMC or monitored by staff for KMC.

The subgroup agreed that the explanation of the kangaroo position for a low birthweight baby, *i.e.* skin-to-skin with a caregiver in an upright position, was clear [9,37]. However, they considered that KMC must be differentiated from routine skin-to-skin contact recommended for all newborns in the first hour after birth [37], compared to KMC, which is a continuous process, recommended for 8–24 hours per day While the subgroup acknowledged the importance of measuring the duration of KMC, it was noted that this aspect primarily reflected a quality measure, rather than coverage. For coverage measurement, it was agreed that ‘initiation’ of KMC was the most practical numerator, consistent with previous work [38]. The subgroup discussed studies that measured other components of KMC, such as breastmilk feeding and post-discharge care plans, but acknowledged that measuring these components routinely could add burden to resource-constrained systems [31,39].

Based on the 2022 updated WHO recommendation of KMC for all preterm (<37 weeks gestation) and/or low birthweight (<2500 g) infants, the subgroup recommended the denominator as the total number of admitted newborns with a birth weight <2500 g [9,40]. They also recommended sub-tabulation for neonates <2000 g where possible. With regard to preterm newborns, the subgroup recognised the challenges with accurately estimating gestational age in many LMICs [41–46], hence the focus on birthweight. However, they noted the importance of building opportunities to improve gestational age assessment by ultrasound in all settings. The subgroup recommended improvements in the quality of weighing techniques after birth. These included using digital weighing scales to mitigate the risk of ‘heaping’ at 2500 g, which might inadvertently exclude babies who could benefit from KMC [47–49], and improved register design [49]. The subgroup also suggested research exploring other potential measures of KMC quality that could be captured in notes or possibly in registers, even if these were not required for reporting in RHIS but for local quality of improvement measures.

### Possible serious bacterial infections – indicator and measurement considerations

Thirteen studies reporting on PSBI indicators were identified from Ethiopia [50,51], Bangladesh [52,53], India [54], Nepal [55], Tanzania [55], Pakistan [55], and Nigeria [55]. These focused on coverage measurement of PSBI antibiotic treatment, adherence to treatment, referrals and follow-ups.

The subgroup found the proposed indicator – the proportion of newborns identified as PSBI in outpatient settings or suspected sepsis in in-patient settings, who receive at least two days of any appropriate injectable antibiotics – to be clear. In 2013 and 2015, WHO guidelines recommended antibiotic therapy for managing PSBI in hospital and non-hospital settings, respectively [56,57]. While this could improve the recording of the numerator, a standardised method for recording receipt of antibiotics (generic name, doses received) for the numerator may be required.

The subgroup acknowledged that diagnosing PSBI, which appears both in the numerator and denominator, requires careful clinical assessments and specific clinical training. They also recognised the limited availability of blood culture to support a diagnosis of PSBI in LMIC settings [57], and proposed improving the availability of blood culture testing could enhance the identification of PSBI.

### Antenatal corticosteroids – indicator and measurement considerations

Only four studies were found that reported on an indicator for ACS measurement, with two studies reporting on the proportion of all preterm newborns whose mothers received at least one dose of ACS [53,58]. No studies were identified that assessed the accuracy of reporting of an ACS indicator.

The subgroup considered the WHO 2022 recommendation on ACS for improving preterm birth outcomes that women at risk of early preterm birth (<34 weeks gestation) receive a total of 24 mg intramuscular steroid in divided doses over 36 hours or until birth, whichever occurs first [9]. However, they concluded that capturing data of multiple doses of ACS into existing data systems would pose challenges in many settings requiring extensive case note review. Therefore, guided by the existing literature, the suggestion of ‘at least one dose’ was supported as the numerator among all women who delivered between 24 and 34 weeks of gestational age (denominator). Such an indicator measures the initiation of the intervention, providing useful information for identifying eligible women for ACS to improve preterm birth outcomes and facilitate access to the intervention, if required.

The WHO only recommends ACS use during the gestational age window of 24 to 34 weeks [9]. Accurate gestational age is an important component of this indicator. Although an early high-quality ultrasonogram would provide the optimal estimate, the subgroup recognised that early ultrasound dating is not always feasible in LMIC settings. Therefore, the group used the term ‘best obstetric estimate’ as a practical compromise, emphasising the need to improve gestational age assessment by ultrasound in all settings. However, the use of postnatal gestational age assessment was discouraged, as it was deemed more beneficial for countries to prioritise improving pregnancy dating through antenatal ultrasound [59].

WHO guidelines also recommend that that preterm birth must be ‘highly likely’ within seven days and that ACS must be used for women at risk of early preterm birth but with no sign infection [9]. The subgroup suggested that a coverage indicator should not be made unnecessarily complex by including these additional recommendations that would be difficult to implement in busy settings. These could be considered as part of indicators reflecting the quality of ACS use at a later stage [9,60], it was important to prioritise the focus on coverage measurement. The subgroup strongly recommended research on ACS indicator measurement in LMIC settings was needed to establish feasibility and accuracy of measurement from case notes and collection in hospital registers.

The expert assessment of the proposed indicators against good indicator criteria are shown in **Table 3**.

**Table 3:** Assessment of the four proposed SSN indicators against the “good indicator” criteria [22]

Criteria	Action-focussed	Important	Measurable	Feasible	Valued	Simple	Accurate
	***It is clear what should be done to improve outcomes associated with this indicator to lead to action to improve quality of care***	***The indicator and data generated will make a relevant, significant contribution to determining how to respond to the health problem effectively.***	***The indicator is quantifiable; the definitions are precise, and reference standards are available and tested or could be developed***	***It will be feasible to collect the data required for the indicator in the relevant setting.***	***The people involved value the indicator***	***The people involved in the service can understand and can use the indicator to improve their work***	***Validated against a standard***
Neonatal Resuscitation	Yes	Yes	Quantifiable, reference range ~ 3%, but may vary with context [26]	Yes, with some changes to registers to capture PPV at birth.	Yes	Yes	Needs testing. Previously tested [18], but with a different denominator.
				Assessing ‘not breathing well’ may require training and skill.			
				Identification of macerated/fresh stillbirths may be challenging.			
KMC	Yes	Yes	Quantifiable, desirable to be as close to 100% as possible. Needs testing.	Yes, with appropriate registers that capture data required at end of an episode of care.	Yes	Yes. Uses a birth weight cut off. Gestational age cut off more challenging	Needs testing. Previously tested [18], but only in KMC ward (not anywhere in facility) and with a different numerator and denominator.
				Will need to be differentiated from skin-to-skin contact at birth.			
				For preterm newborns, accurate antenatal gestational age assessment can be challenging in some contexts.			
				Requires accurate weighing at birth.			
Antibiotic treatment for neonatal infection	Yes	Yes	Quantifiable, but requires training. Reference range variable by context.	Yes, if there are processes to capture the numerator and denominator from individual records into registers.	Yes	Has a complex/numerator and denominator	Needs testing with proposed numerator and denominator. Not tested against register recorded (previously only against survey questions) [18].
				Clinical training/skills to diagnose sepsis required.			
				Standardised method of recording antibiotic use needed.			
				Increasing access to blood culture facilities could help better diagnosis			
ACS use	Yes	Yes	Well defined, but no testing has been done.	Yes, if there are processes to capture the numerator and denominator from individual records into registers.	Yes	Has a complex/numerator and denominator	No previous validation
				For preterm newborns, accurate antenatal gestational age assessment can be challenging in some contexts.			

ACS – antenatal corticosteroid, KMC – kangaroo mother care, PPV – positive pressure ventilation

## DISCUSSION

The purpose of this WHO-led consultation was to convene a multidisciplinary and institutionally diverse group of experts to discuss and agree on definitions of SSN indicators for management of complications that could be most feasibly collected, reported, and monitored in an RHIS. Challenges were identified, the most common being the need to extract information from case records at the end of an episode of care. Testing of feasibility of collection, ways to improve data collection in different contexts for the proposed indicators is required.

In high mortality settings, reporting and monitoring SSN interventions are important not only to enhance care within facilities, but also to support programme managers and policy makers in planning health services to improve newborn survival. These are settings where the collection of information for these indicators is also likely to be the most challenging.

Improved data quality and continuous use of information in health systems have been reported to be affected by three categories of determinants: technical, behavioural and environmental/ organisational [61].

### Technical challenges

Currently, most data from high mortality settings are aggregated from routine registers. In some instances, these registers may capture neonatal resuscitation since this intervention typically occurs once immediately after birth. However, registers are not the optimal data source for the other SSN interventions of KMC, PSBI and ACS which occur over the course of an inpatient episode. These indicators rely on individual case notes as source data, but the data quality of case notes data in LMICs may vary [62]. Incomplete documentation in non-standardised case notes could lead to poor indicator capture and reduced accuracy. Structured standardised clinical case sheets could facilitate the collection of the data necessary for these indicators [63]. Recent advances in digital platforms are transforming RHIS in many high mortality settings. However, in many high mortality settings, paper-based registers and individual case notes are currently the source data entered to digital data platforms and are likely to remain so for the coming decade.

### Organisational challenges

It is important to develop a ‘data culture’ in the facility around maternal and newborn care. To improve the feasibility of collecting and reporting these indicators, it is essential to ensure health workers have the necessary time, training, supervision, capacity and motivation to carefully document the care they provide in medical records, whether in registers or clinical case notes. Enhancing health workers’ understanding of the data’s significance for service monitoring and improvement within the facility can foster their active engagement in the process [63]. Decentralised data use in labour wards and newborn units may incentivise improving data quality [64,65]. It has been shown that data quality in registers can be improved by increasing data visibility through feedback to frontline health workers about data use and data quality [66,67]. Given that the indicators for SSN care are pertinent to only to a small proportion of cases, securing buy-in from heath workers is crucial to facilitate the accurate recording of these indicators. Extraction of data from case records into registers could be facilitated by using structured case sheets which include the parameters to be recorded.

Besides embedding a data culture and data entry, use and quality can also be enhanced by supportive supervision of staff. Streamlined data processes (including the use of structured case sheets) will reduce the burden on staff, while incentives for staff, including opportunities to present the data, and the provision of positive feedback will encourage staff and help embed a data culture.

The various physical locations where different elements of care are provided and recorded (*e.g.* ACS in the antenatal ward, antibiotics in the sick newborn unit) also create additional coordination requirements for data collection [68]. The cumulative effect of distance between point of care and point of register documentation, simultaneous responsibilities of care and documentation for a large number of data elements to be recalled has been shown to affect the reporting of interventions [23].

### Behavioural challenges

Well-trained and motivated obstetric and neonatal unit staff, with access to constructive supervision and support for problem-solving when such situations arise during data collection and recording, are important to ensure the quality of data [61]. Demand at the facility level to track coverage of these SSN indicators to improve care, besides national/ regional demand for planning, will also support the embedding of these indicators and improve the quality of the data collected.

### Improving measurement and reporting of key indicators for SSN care

As discussed, a number of challenges exist to the measurement of reporting of indicators for these four lifesaving interventions, particularly in resource-constrained, high neonatal mortality contexts. Besides the specific technical research needs identified in relation to each indicator, a further exploration of possible barriers and facilitators to collecting the information necessary to contribute towards these four key indicators is required. A clear understanding of the barriers will allow the development of potential ways in which they can be surmounted. These potential solutions can then be tested for feasibility in obstetric and neonatal units in different contexts.

Simultaneously, as feasibility for collection and reporting of the SSN care indicators is being tested through implementation research, it is also important that departments of health and donors promote a ‘data culture’ in the facility. It is important that staff are aware of the importance of data collection for improving newborn survival. They should be able to use the data to track the provision of these services at facility level to plan action locally and improve services in their facility. It is also important for stakeholders to support data collection for these indicators of SSN care as a planned, purposeful activity, with resources committed towards these actions. Departments of health already prioritise the reduction of neonatal mortality. It is important for departments to view the provision of these four interventions as a significant contributor to a reduction in neonatal mortality, and therefore prioritise and necessitate the reporting of these indicators in RHIS. The reporting of these indicators will be challenging, and it could take some time, depending on context, to streamline processes resulting in good quality indicators for SSN care being reported. Focussing on strengthening the measurement of these four indicators is likely to also result in an overall improvement in the reporting of other maternal newborn indicators reported under ENAP-EPMM.

These four indicators are intended to measure the ‘coverage’ of these four services, *i.e.* the number of newborns receiving each of the four services (numerator) from among all newborns in need of the service (denominator). However, during the consultation, the need for indicators to measure the quality of each service was also discussed. While these are not included in the 10 core ENAP-EPMM indicators, such indicators would measure different domains of coverage (*e.g.* timing, completion rates, safety) for selected interventions; for example, for KMC, one such indicator could include the proportion of babies receiving KMC, for eight hours or more, during the last 24 hours. While these indicators are important, the focus of this consultation has been on the coverage of four key SSN intervention indicators.

### Planning for integration in an RHIS

The proposed indicators, once tested for feasibility, will need to be reported into RHIS. At the point of service delivery, structured individual case notes will be the basis of data for these indicators. As discussed above, structured notes will facilitate the extraction of the necessary elements of each indicator for entry into registers at the facility level. The collated data and indicators at the facility level are useful to inform planning decisions and local quality improvement, including audits. Facility-level data on coverage of SSN are intended to be aggregated at the district level and subsequently at the national level. At these levels, the information will help planning (*e.g.* human resources, equipment and drug availability).

It is important for accountability purposes to track a few core, standardised indicators closely related to maternal and newborn survival. ENAP has prioritised 10 core indicators, of which four include coverage of the four key SSNC services. The WHO has provided recent guidance on strategies for optimising national RHISs to deliver universal health coverage [69], which includes care for SSN. There are examples of countries that have successfully begun the recording of these key indicators for SSNC into RHIS. Malawi’s Ministry of Health, Reproductive Health Directorate and Central Monitoring and Evaluation Department has developed a national routine reporting system for KMC, including a simplified, user-friendly KMC register and reporting form. National rollout in 2015 led by the Central Monitoring and Evaluation Department showed 87% of hospitals reported on KMC [33]. Similarly, the National Health Mission in India, implements one of the largest online databases of small and sick newborns globally, covering 2.7 million newborns across units across the country. This database captures real-time data for a large number of parameters, including admission profile, final diagnosis, and treatment parameters, and could thus easily be adapted to provide the coverage of these four SSNC services [70].

Understanding, improving, and sustaining equity in coverage is a key component of the WHO vision for maternal, newborn, and child health [71]. It is recommended that the ENAP core indicators for coverage of care for newborns at risk or with complications be tracked in such a way that they can be broken down to assess equity in access to these services, *e.g.* urban or rural, regional, wealth quintile, so that steps can be taken to reduce inequities in access to SSN care [72].

## CONCLUSION

The proportion of births occurring in health facilities across the world has been steadily increasing. This trend has been accompanied by recent efforts to scale up inpatient SSN care, including the provision of the four SSN interventions in high neonatal mortality settings. This consultation defined four key indicators, one for each key intervention for SSN. While the measurement of these indicators is challenging, it is important that their progress is tracked both at facility level and ultimately in RHIS. There is a clear need for testing and finding innovative ways to support the feasibility of the measurement of these indicators in different health system contexts. A ‘data culture’ needs to be supported in obstetric and neonatal units, which is planned, resourced and allows for capacity building, supportive supervision, and use of the data collected at the level of collection. Simultaneously, it is important that departments of health demand tracking the progress of these key interventions at national level, given their relationship to the reduction of neonatal mortality. While acknowledging the challenges to collect these indicators for RHIS, it is clearly important that efforts are made to measure and track progress on the delivery of these four life-saving interventions, which are critical for country progress towards SDG 3.2.

## Additional material


Online Supplementary Document
